# Plane-Based Sampling for Ray Casting Algorithm in Sequential Medical Images

**DOI:** 10.1155/2013/874517

**Published:** 2013-01-22

**Authors:** Lili Lin, Shengyong Chen, Yan Shao, Zichun Gu

**Affiliations:** ^1^School of Computer Science and Technology, Zhejiang University of Technology, Hangzhou 310023, China; ^2^Department of Plastic and Reconstructive Surgery, Sir Run Run Shaw Hospital, Medical College, Zhejiang University, Hangzhou 310016, China

## Abstract

This paper proposes a plane-based sampling method to improve the traditional Ray Casting Algorithm (RCA) for the fast reconstruction of a three-dimensional biomedical model from sequential images. In the novel method, the optical properties of all sampling points depend on the intersection points when a ray travels through an equidistant parallel plan cluster of the volume dataset. The results show that the method improves the rendering speed at over three times compared with the conventional algorithm and the image quality is well guaranteed.

## 1. Introduction

Modeling three-dimensional (3D) volume of biomedical tissues from 2D sequential images is an important technique to highly improve the diagnostic accuracy [1]. Volume rendering refers to the process that maps the 3D discrete digital data into image pixel values [2]. It can be classified into two categories: one is direct volume rendering which generates images by compositing pixel values along rays cast into a 3D image, and the other one is indirect volume rendering which visualizes geometry element graphics extracted from the volume data [3]. The importance of volume rendering is resampling and synthesizing image [4]. Ray casting, splatting, and shear-warp are the three popular volume rendering algorithms now [5]. 

Ray Casting Algorithm (RCA) is a direct volume rendering algorithm. The traditional RCA is widely used for it can precisely visualize various medical images with details of boundary and internal information from sequential images, while real-time rendering with traditional RCA is still an obstacle due to its huge computation.

In recent years, numerous techniques have been proposed to accelerate the rendering speed. In general, there are three primary aspects, including hardware-based, parallel, and software-based acceleration algorithms. Liu et al. [6] proposed a method combined that Graphics Processing Unit (GPU) and octree encoding and accelerated RCA at a rate of 85 times. Wei and Feng [7] presented a GPU-based real-time ray casting method for algebraic B-spline surfaces via iterative root-finding algorithms. Zhang et al. [8] accelerated RCA on Compute Unified Device Architecture (CUDA), which can perform more samplings within a ray segment using cubic B-spline. 

However, both hardware-based and parallel techniques are inseparable from the development of computer hardware. By comparison, software-based algorithms can be quickly transplanted among different machines. What is more, they can show flexibility of the procedure and reflect the thoughts of researchers. Yang et al. [9] sampled points based on all intersection points at which the ray transacts with the voxel. All intersections in a voxel depend on four vertexes on one face. However, the condition whether two intersection points were on adjacent or opposite surface in a voxel was neglected. Ling and Qian [10] used a bounding volume method to avoid casting the viewing rays that do not intersect with the volume. Since such situation can be judged quickly by comparing the world coordinates of sampling point with the volume dataset, it did not obviously speed up the rendering process. Recently, Qian et al. [11] replaced the sampling points with intersection points when rays travel through three groups of parallel planes along three orthometric axes to reduce the rendering time. However, it cannot guarantee the image density when the distance between adjacent parallel planes far surpasses the sampling interval.

This paper proposes an improved RCA to speed the rendering process. The main idea is, when the ray travels through one group of equidistant parallel planes of the volume, intersection points are obtained. Then the properties of sampling points between adjacent intersection points can be calculated by the formula of definite proportion and separated points. By this method, a small number of intersection points are considered; meanwhile the method does not sacrifice the sampling density.

## 2. Ray Casting Algorithm

### 2.1. Ray Casting Algorithm Overview

The traditional RCA involves two steps: (1) assign optical properties such as color and opacity to all 3D discrete vertexes according to their gray value, and (2) apply a sampling and composing process. For each output image pixel in sequence, do the following.

(i) Cast the ray through the volume from back to front.
(ii) Sample the color *c*_*i*_ and opacity *a*_*i*_ at each regular sampling point along the ray.
(iii) Set the color of the current output pixel according to
  (1) cout=∑i=0n−1c(i)∏j=0i−11−a(j)=c0+c1(1−a0)+c2(1−a1)(1−a0)+⋯.

The rendering time is mainly comprised of four parts in the above-mentioned rendering process [11]. They are converting gray value into optical property (about 30%), computing position of sampling points (about 3%), sampling optical properties (about 39%), and compositing properties into output pixel color (about 6%). The time proportion of sampling is the highest. Moreover, the time ratio of four parts is not constant. The greater the sampling data is, the larger the proportion of sampling time is. Therefore, sampling has a direct impact on speed of RCA.

### 2.2. Traditional Sampling Method

**Figure 1:**
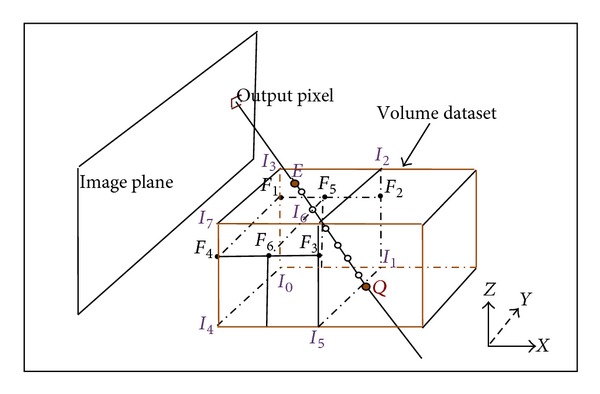
Interpolation for ray casting.

Traditionally, the optical property of each sampling point depends on eight vertexes of its voxel by trilinear interpolation [12, 13]. In detail, there are four steps for the sampling one point. First, locate its voxel and convert the world coordinates of sampling point into voxel's local coordinates. The following three steps are processes of linear interpolations along three different axes in order. The interpolation diagram of Ray Casting Algorithm is shown in Figure 1.

For example, to sample point *S*(*x*, *y*, *z*) in white circle (Figure 1), first obtain the voxel (*i*, *j*, *k*) and local coordinates (*x*_*n*_, *y*_*n*_, *z*_*n*_) of *S*, which are expressed in (2). Then the optical property of four points (*F*_1_, *F*_2_, *F*_3_, *F*_4_) on the plane through *S* is deduced according to eight vertexes (*I*_0_ ~ *I*_8_) along *z*-axis. The next property of two points (*F*_5_, *F*_6_) forming the line segment through *S* is computed along *x*-axis. At last *S* is obtained along *y*-axis by definite proportional division point formula.

In Figure 1, assume the pixel spacing along *x*-, *y*-, *z*- axes is Δ*x*, Δ*y*, and Δ*z*, respectively, with *I*_0_(*x*_*i*_, *y*_*j*_, *z*_*k*_):

(2) $$\begin{array}{c} i=\left[\frac{x}{\Delta x}\right],\;\;j=\left[\frac{y}{\Delta y}\right],\;\;k=\left[\frac{z}{\Delta z}\right],\\ x_{i}=i\times \Delta x,\;\;y_{j}=j\times \Delta y,\;\;z_{k}=k\times \Delta z,\\ x_{n}=\frac{x-x_{i}}{\Delta x},\;\;y_{n}=\frac{y-y_{j}}{\Delta y},\;\;z_{n}=\frac{z-z_{k}}{\Delta z}, \end{array}$$

where operator [·] represents taking the floor integral.

The property *F* of *S* can be calculated by *F*_5_ and *F*_6_, which are obtained by *F*_1_, *F*_2_, *F*_3_, and *F*_4_. The relationship between them is shown in

(3) $$\begin{array}{c} F_{1}=I_{0}+z_{n}\times \left(I_{3}-I_{0}\right),\;\;F_{2}=I_{1}+z_{n}\times \left(I_{2}-I_{1}\right),\\ F_{3}=I_{5}+z_{n}\times \left(I_{6}-I_{5}\right),\;\;F_{4}=I_{4}+z_{n}\times \left(I_{7}-I_{4}\right),\\ F_{5}=F_{1}+x_{n}\times \left(F_{2}-F_{1}\right),\;\;F_{6}=F_{4}+x_{n}\times \left(F_{3}-F_{4}\right),\\ F=F_{5}+y_{n}\times \left(F_{6}-F_{5}\right). \end{array}$$

According to the above equations, 17 additions and 16 multiplications are executed for sampling each point such as *S* (see Figure 1), including 3 additions and 9 multiplications to locate the voxel (*i*, *j*, *k*) and get the local coordinates. In Figure 1, there are 6 sampling points in two voxels, 102 additions, and 96 multiplications performed. To simplify the calculation of sampling process, a new RCA based on plane clusters sampling is proposed.

### 2.3. Proposed Plan-Based Sampling Method

The basic idea of the plan-based sampling method is to acquire all sampling points based on intersection points when ray travels through a group of parallel planes in the volume data field. 

The sampling process, specifically, consists of three steps. First, intersections and the corresponding plane are obtained based on some necessary initial conditions. Then the optical property of all the intersection points is obtained by linear interpolation according to vertexes on plane clusters. The optical property of sampling points between intersection points along the ray is computed by definite proportion and separated point formula.

Assuming that the direction vector of ray is *ζ* = (*r*, *m*, *n*) and the extent of gridding volume data is *Ex* × *Ey* × *Ez*, with the spacing Δ*x*, Δ*y*, Δ*z* along *x*-, *y*-, *z*- axes, respectively, the three plane clusters are as follows:

(4) $$\begin{array}{c} X_{i}=i\Delta x\;\left(i=0,1,2,\ldots ,Ex-1\right),\\ Y_{j}=j\Delta y\;\left(j=0,1,2,\ldots ,Ey-1\right),\\ Z_{k}=k\Delta z\;\left(k=0,1,2,\ldots ,Ez-1\right). \end{array}$$

Parallel plane clusters along *y* axis are selected. Let the origin point of ray be *O*(*x*_*o*_, *y*_*o*_, *z*_*o*_). The ray intersects with plane *Y*_*j*_ at entry point *E*(*V*_*i*_, *V*_*j*_, *V*_*k*_) and *E* belongs to the voxel (*i*, *j*, *k*). The coordinates of *E* and voxel (*i*, *j*, *k*) are deduced next. The derivation is shown as follows. Since

(5) $$\begin{array}{c} V_{j}=y_{o}+m\times t_{j}=j\Delta y\;\left(j=0,1,2,\ldots ,Ey-1\right), \end{array}$$

where *t*_*j*_ means the distance from *O* to *E* along ray, the value of *j* can be obtained from

(6) $$\begin{array}{c} j=\left[\frac{y}{\Delta y}\right]. \end{array}$$

Therefore,

(7) $$\begin{array}{c} t_{j}=\frac{j\Delta y-y_{o}}{m}, \end{array}$$

and *V*_*i*_, *V*_*k*_ *of E*(*V*_*i*_, *V*_*j*_, *V*_*k*_) can be expressed as follows:

(8) $$\begin{array}{c} V_{i}=x_{o}+r\times t_{j};\;\;V_{k}=z_{o}+n\times t_{j}. \end{array}$$

Considering that *E* belongs to voxel (*i*, *j*, *k*), then *i* and *k* are expressed as follows:

(9) $$\begin{array}{c} i=\left[\frac{V_{i}}{\Delta x}\right],\\ k=\left[\frac{V_{k}}{\Delta z}\right]. \end{array}$$

Therefore, when *j*is given, *E*(*V*_*i*_, *V*_*j*_, *V*_*k*_), *i* and *k* can be obtained through the above equations.

From the mathematical derivation, when original position, direction vector, and the extent of volume data are given, all the intersections and associated voxels can be quickly obtained.

In Figure 1, the property *I*_*E*_ of entry point *E* can be computed by the property (*I*_0_, *I*_1_, *I*_3_) of three vertexes on voxel (*i*, *j*, *k*), that is,

(10) $$\begin{array}{c} I_{E}=I_{0}+\left(I_{1}-I_{0}\right)\left(\frac{V_{i}}{\Delta x}-i\right)+\left(I_{3}-I_{0}\right)\left(\frac{V_{k}}{\Delta z}-k\right). \end{array}$$

In the same way, the property *I*_*Q*_ of exit point *Q* can be obtained. At last the property *I*_*S*_ is expressed as follows:

(11) $$\begin{array}{c} I_{S}=I_{E}+\frac{t-t_{j}}{t_{j+1}-t_{j}}\left(I_{Q}-I_{E}\right). \end{array}$$

In addition, when one component of the direction vector **ζ** is zero, a plane cluster along another axis can be chosen. If two components are zero, the plane clusters along the third axis are taken into account.

### 2.4. Comparison of Two Sampling Methods

In the new RCA sampling process, only intersection points on a plane cluster along one axis need to be considered without converting coordinates. While in the conventional sampling process, the world coordinates of each sampling point are converted into voxel's local coordinates and computed by trilinear interpolation [14, 15]. 

As is shown in Figure 1, there are 6 sampling points between *E* and *Q*. 15 additions and 19 multiplications are executed to sample *E* and *Q*, and 24 additions and 12 multiplications are run to sample six points based on *E* and *Q*. Totally, 39 additions and 31 multiplications are taken compared with 102 additions and 96 multiplications with trilinear interpolation. Furthermore, not all vertexes are referred because some vertexes (such as *I*_4_, *I*_7_, *I*_2_ in Figure 1) are not used as reference by the new method. Thus, in theory, the calculation amount is reduced to less than one third on the whole.

## 3. Experiments and Analysis

### 3.1. Data

Experiments are carried out on head CT sequences and heart CT sequences. Both sequences are scanned by Siemens spiral CT. The detail information is shown in Table 1. Taking head for an example, the extents are 512 × 512 × 295, and the pixel spacing is 0.486 mm, 0.486 mm and 0.700 mm along *x*-, *y*-, *z*- axis, respectively. The sampling distance along ray is 0.3 mm.

### 3.2. Results

**Figure 2:**
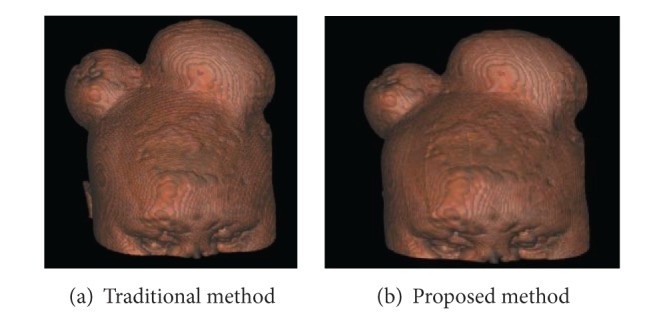
Head images of ray casting.

**Figure 3:**
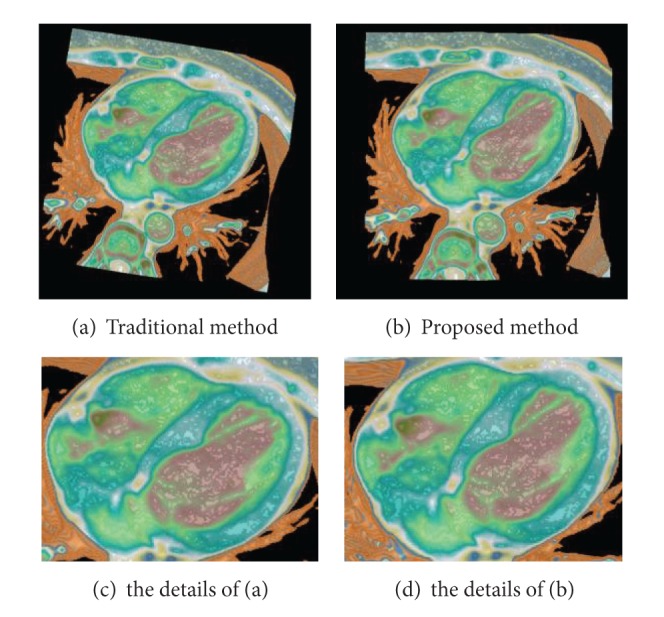
Heart images with ray casting.

The reconstructed results of two datasets are shown in Figures 2 and 3. The rendering time of the data is shown in Table 1. For example, it takes 17.158 seconds to render the head sequences with the new sampling method, while 58.274 seconds using the traditional method.

### 3.3. Analysis

The new sampling method does not consult all 3D vertexes of the volume data. For this reason, it is a question whether the image quality can be guaranteed. It can be seen in Figures 2 and 3 that images reconstructed by RCA based on plan cluster sampling method are almost the same as those based on traditional trilinear interpolation in RCA. They can clearly show the details of the boundary and internal information of the volume with the new sampling method. Therefore, the image quality can be well ensured.

By comparing the amount of computation (39/102-31/96) in the two sampling methods, the new method can reduce the amount of traditional one to about one third. It can be seen that the total rendering time (Table 1) using new method is less than one third of that using conventional trilinear interpolation. It indicates that the time saved to inquire the property of the vertexes not for reference should not be underestimated.

Moreover, it is shown that the acceleration rate of the head images is higher than that of the heart images. The main difference between them is that the spacing of head CT sequences is denser than the heart data. Therefore, the denser the data is, the more efficient the new method is.

## 4. Conclusion

This paper presented a novel RCA based on a parallel plan cluster sampling method. The proposed method can efficiently speed up the sampling process at more than three times and still clearly display the boundary and internal information of the volume; thus the image quality is well guaranteed. In addition, the comparison of acceleration rate indicates that the new method is more effective for dataset with denser spacing. The new method can meet the real-time requirements of interactive rendering.

## Figures and Tables

**Table 1:** Comparison of two sampling methods.

Objects and sizes	Head 512 × 512 × 295	Heart 512 × 512 × 41
Spacing (mm × mm × mm)	0.486 × 0.486 × 0.700	0.318 × 0.318 × 2.000
Sampling distance (mm)	0.3	0.3
Time by the traditional (s)	58.274	7.192
Time by the proposed (s)	17.158	2.043
Acceleration rate	3.606	3.52
